# Validation of snoring detection using a smartphone app

**DOI:** 10.1007/s11325-021-02359-3

**Published:** 2021-04-03

**Authors:** Jui-Kun Chiang, Yen-Chang Lin, Chih-Wen Lin, Ching-Shiung Ting, Yi-Ying Chiang, Yee-Hsin Kao

**Affiliations:** 1 Department of Family Medicine, Dalin Tzu Chi Hospital, Buddhist Tzu Chi Medical Foundation, 2, Minsheng Road, Dalin, 62247 Chiayi Taiwan; 2 Nature Dental Clinic, 341, Sec. 3, Zhongshan Rd, Puli Township, Nantou 545 Taiwan; 3 Departments of School of Medicine, Tzu Chi University, Hualien, Taiwan; 4 Department of Medical Imaging, Dalin Tzu Chi Hospital, Buddhist Tzu Chi Medical Foundation, Chiayi, Taiwan; 5 Department of Otolaryngology, Tainan Municipal, 670 Chung-Te Road, Tainan, 70173 Taiwan; 6 Department of Family Medicine, Tainan Municipal Hospital (Managed by Show Chwan Medical Care Corporation), 670 Chung-Te Road, Tainan, 70173 Taiwan

**Keywords:** Snoring, Snore Clock, Smartphones, App

## Abstract

**Purpose:**

Snoring is closely related to obstructive sleep apnea in adults. The increasing abundance and availability of smartphone technology has facilitated the examination and monitoring of snoring at home through snoring apps. However, the accuracy of snoring detection by snoring apps is unclear. This study explored the snoring detection accuracy of Snore Clock—a paid snoring detection app for smartphones.

**Methods:**

Snoring rates were detected by smartphones that had been installed with the paid app Snore Clock. The app provides information on the following variables: sleep duration, snoring duration, snoring loudness (in dB), maximum snoring loudness (in dB), and snoring duration rate (%). In brief, we first reviewed the snoring rates detected by Snore Clock; thereafter, an ear, nose, and throat specialist reviewed the actual snoring rates by using the playback of the app recordings.

**Results:**

In total, the 201 snoring records of 11 patients were analyzed. Snoring rates measured by Snore Clock and those measured manually were closely correlated (*r* = 0.907). The mean snoring detection accuracy rate of Snore Clock was 95%, with a positive predictive value, negative predictive value, sensitivity, and specificity of 65% ± 35%, 97% ± 4%, 78% ± 25%, and 97% ± 4%, respectively. However, the higher the snoring rates, the higher were the false-negative rates for the app.

**Conclusion:**

Snore Clock is compatible with various brands of smartphones and has a high predictive value for snoring. Based on the strong correlation between Snore Clock and manual approaches for snoring detection, these findings have validated that Snore Clock has the capacity for at-home snoring detection.

## Introduction

Snoring, defined as “the vibration of palatal soft tissue from obstruction of air movement on breathing during sleeping,” is believed to be a key indicator of possible obstructive sleep apnea syndrome (OSA) [1]. Snoring prevalence in Asian countries, in descending order, is Taiwan (59%) [2], Malaysia (47%) [3], Turkey (41%) [4], Japan (24% among men and 10% among women) [5], Singapore (7%) [6], and Thailand (5%) [7]. Snoring prevalence also differs among ethnicities. The higher snoring prevalence in Chinese descents compared with Caucasians might be due to Chinese people having narrower cranial bases and flatter mid-face structures [8].

OSA is closely associated with several serious illnesses, including systemic hypertension, coronary artery disease, stroke, and metabolic syndrome; it may also result in motor vehicle accidents and diminished quality of life [9–11]. OSA is insidious, and patients are often unaware of the associated symptoms. The cardinal manifestations of OSA include loud snoring, breathing pauses during sleep, fitful sleep quality, and excessive daytime sleepiness [12]. Although not all patients with snoring have clinically significant sleep apnea, snoring is the earliest and most common symptom of OSA, occurring in 70–95% of patients with OSA [13, 14]. In addition, snoring intensity increases with OSA severity [14], and snoring itself can present a vascular risk [15].

One related study distinguished between two types of snore: the first type has a very low frequency (<20 Hz) but large-amplitude vibrations lasting from 0 to 1 s and 2.5 to 4.5 s; and the second type involves high-frequency sounds with small-amplitude vibrations lasting from 1.0 to 1.5 s and 4.5 to 5.0 s [15]. Another study reported that snores can be categorized into irregular and regular snores and noted that patients with severe OSA had a shorter interval between regular snores [16]. Various obstruction sites also induce different snoring frequencies. Common obstruction sites include the velum, oropharynx, tongue base, and epiglottis [17]. The frequencies of more severe snoring differ considerably from those of habitual snoring, which range from 110 to 190 Hz [18, 19]. However, in patients with OSA, the snoring frequency might be higher than 800Hz [20, 21]. Notably, the average snoring sound intensity is higher for men than for women [19]. Polysomnography (PSG) is the gold standard method for diagnosing OSA and monitoring snoring [13]. However, PSG must be performed at special sleep treatment centers where patients sleep overnight. Patients undergoing PSG may experience some inconveniences. First, many patients do not sleep well during PSG examination because of the discomfort resulting from the cumbersome monitoring wires required for this test. Second, patients might face wait times of several weeks or even longer for a PSG examination in Taiwan. Third, the PSG test is a time-consuming procedure that can result in considerable patient discomfort [22]. Therefore, simplified recording and monitoring instruments that can conveniently and reliably diagnose snoring and OSA at home are necessary [23]. A related study suggested that sleep physicians consider repeat testing with home sleep apnea tests (HSATs) in patients with a negative PSG and clinical symptoms of OSA [24]. HSAT devices are classified by the United States Food and Drug Administration (FDA) as Class II medical devices, which the FDA defines as “devices for which general controls are insufficient to provide reasonable assurance of the safety and effectiveness of the device” [25]. However, HSATs have some limitations. For example, mispositioned pulse oximeters can yield false low readings [26]. HSAT devices that include cardiorespiratory portable monitors have sensors that can detect breathing patterns and pulse oxygen saturation, masks with tubes for insertion into patients’ nostrils, and sensors for patients’ abdomen and chest. These devices may interrupt sleep and be inconvenient for most patients, who may be unable to afford the cost of using HSAT devices at home every night. Finally, raw data from HSAT devices cannot be read directly and must be reviewed and interpreted by a certified physician.

Along with advances in science and technology, the functions of smartphones have expanded dramatically and rapidly. In addition to their communication features, smartphones allow users to download apps through online stores. More than 100,000 health apps are available on the Android and iOS platforms, with one target for health and fitness apps being sleep and sleep hygiene [27]. Snoring apps are software applications that run on smartphones and record sound information while the user sleeps; such apps have been available for several years and enable convenient and personalized sleep care [28]. Snoring apps are undoubtedly a simple and feasible option for monitoring snoring. With the progress of innovative monitoring mechanisms and techniques, these apps can be used regularly at home for examinations without interrupting a person’s sleep. The precision of smartphone apps for predicting snoring reportedly ranges from 93 to 96%, with a sensitivity ranging from 64 to 96%; however, app performance can vary greatly among smartphone models [29]. This study explored snoring detection accuracy in one of these smartphone apps, Snore Clock.

## Materials and methods

### Patients and methods

Patients with snoring problems using the Lin oral appliance (I602555 [Taiwan], ZL 2013 1 0753192.9 [China]) [30] were recruited from a dental clinic. The inclusion criteria were patients with snoring problems who volunteered to participate in this study. The patients independently downloaded the paid app SnoreClock. Patients aged <20 years were excluded from the study. All patients placed their smartphones within arm’s reach just before falling asleep. Smartphones were positioned on the upper side of the shoulder within 30 cm of the head for optimal recording of snoring sounds. However, no special restrictions with respect to placing the phone on the bed or on a bedside table were applied. The patients recorded their snoring using SnoreClock, following their natural sleep habits for 2–3 weeks, after which the smartphones were returned to the researchers.

### Snore Clock

Snore Clock provides the following data on the duration and intensity of snoring: sleep duration, snoring duration, snoring loudness (dB), maximum snoring loudness (dB), and rate of snoring duration (%). Snore Clock also has a feature that enables users to focus on particular snoring events to facilitate playback of the most notable snoring sounds. This app is available for both iOS and Android devices. The author of Snore Clock was not involved in this study.

All the recordings from Snore Clock were replayed and verified. Snoring was defined as snorting or grunting sounds while asleep. If Snore Clock displayed signals with no spiking waves, snoring was likely to be absent. However, segments of spiking waves indicated a higher possibility of snoring. The app marked snoring periods (including snoring signals and pauses) with red bands and calculated the rate of total snoring duration for all recording sessions.

Before we determined the presence of snoring signals, we conducted a test: the snoring of a randomly selected case (28-year-old man; body mass index = 23.3) was recorded simultaneously with a digital sound recorder using linear pulse-code modulation (ICD SX-2000, Sony Electronics Inc., Tokyo, Japan) and Snore Clock during sleep. The sound recorder had two built-in high-performance dynamic microphones. We set the low-cut filter switch to “OFF” in order to obtain snoring and breathing sounds, and we set the sampling rate to 44,100 Hz. We analyzed the dominant frequencies every 0.01 s. We carefully listened to this recording on the computer and compared the results between the digital recorder and SnoreClock. We noted that interrupted sounds with a large volume (higher dB in the app) were easily recognized as snoring by the app. These noisy sounds included signals with low frequencies (<1000 Hz) or high frequencies (wide frequency bands, as high as 15,762 Hz). However, sounds with smaller volumes with frequency spectrograms resembling snoring sounds on the computer were generally neglected by this app. These sounds were similar to stronger expiration air flows on the smartphone (frequencies < 5000Hz). All these processes required >10 h to complete.

We then carefully listened to the recordings; any equivocal sounds were confirmed by an ear, nose, and throat (ENT) specialist (Ching-Shiung Ting, M.D.): this is referred to as the “manual method” hereafter. The snoring epoch is composed of several snoring signals interrupted by pauses (<10 s) [31]. Therefore, the duration of snoring was represented by the sum of total snoring epochs.

The study protocol was reviewed and approved by the Research Ethics Committee of the Buddhist Dalin Tzu Chi Hospital in Taiwan (No. B10703013).

### Statistical analysis

Statistical software R (version 3.6.1) was used for all statistical analyses. Statistical significance was set at *p* < 0.05, and all tests were two-tailed. Categorical variables are presented as frequency and percentage, and continuous variables are presented as mean ± standard deviation. Univariate linear regression analyses and correlations were performed to evaluate the factors associated with snoring rates. The sensitivity, specificity, positive predictive value, and negative predictive value of the app were calculated. Analysis of variance (ANOVA) was performed to test the mean differences between groups.

## Results

In total, 220 recordings from 11 patients were collected. We excluded recordings of <5 h (19 recordings) and finally included 201 recordings for further analysis. Three Asus ZenFone Max Plus (M1), three Apple iPhone 6, three Sony Xperia, one HTC One, and one Xiaomi Redmi 5 were used for the recordings. Short recordings (<5 h) were excluded because they might be reflective of patients with poor sleep or insufficient phone battery power. The patients’ basic demographic data are presented in Table 1. No patient had a history of diabetes, respiratory disease, sleep disorders, or alcohol or smoking problems.

**Table 1:** Basic demographic characteristics of subjects

Variables	*n* = 11
Gender (male/female)	7/4
Age, years	38.2±11.9
Body mass index	23.6±3.2
Hypertension history, yes	1

**Fig. 1:**
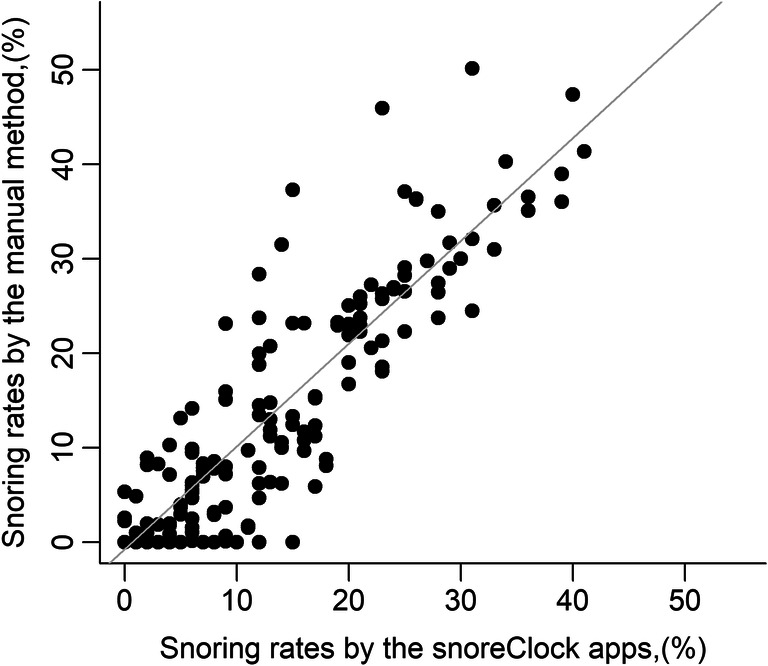
Scatter plot of snoring rates determined by Snore Clock and a manual method of measurement

**Fig. 2:**
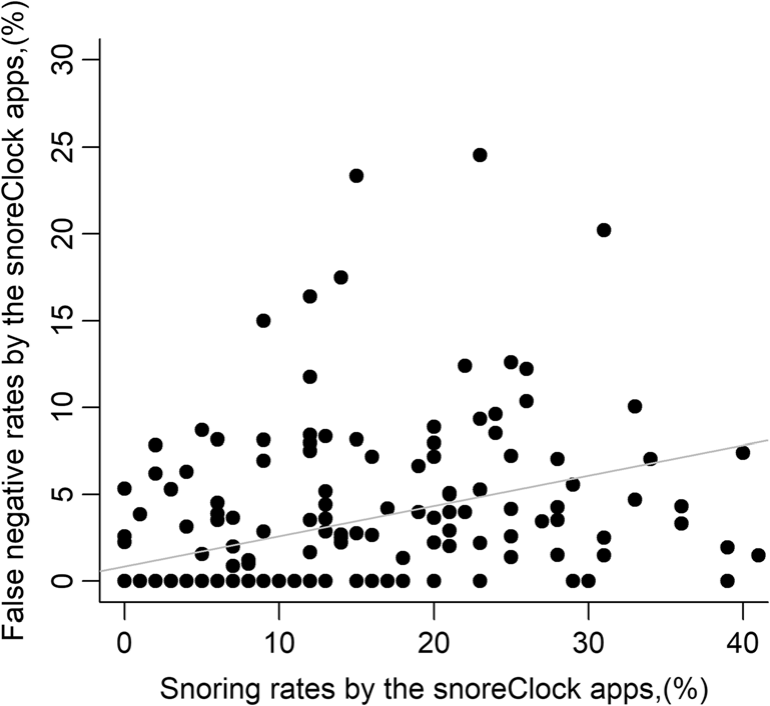
Scatter plot of the false-negative snoring rates by Snore Clock vs. the rates by Snore Clock

**Fig. 3:**
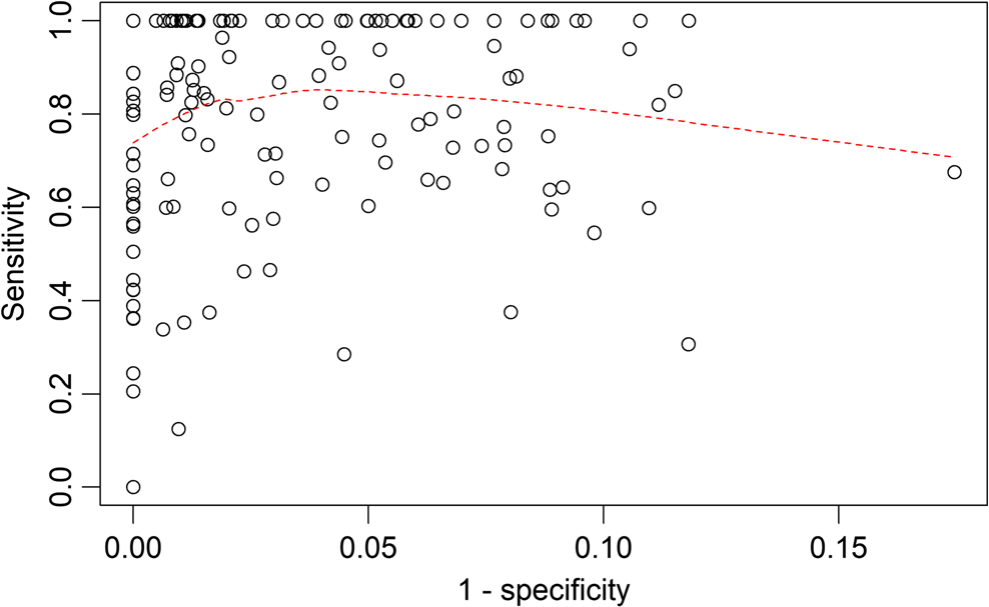
Scatter plots of sensitivities versus 1 – specificities and the smoothing line

The mean accuracy rate for snoring detection by SnoreClock was 95% (comprising 9% snoring rate and 86% non-snoring rate, approved both by the app and manual method). The mean snoring rates calculated by SnoreClock were only 0.3% less than those calculated using the manual method (11.3% ± 10.8% vs. 11.6% ± 12.9%; Table 2). The snoring rates measured by SnoreClock were highly correlated with the snoring rates measured using the manual method (*r* = 0.907, *β* = 1.09, *p* < 0.001; Fig. 1). Moreover, the false-negative rates for SnoreClock positively correlated with the false-negative rates for the manual method (*r* = 0.431, *β* = 1.06, *p* < 0.001; Table 3 and Fig. 2). The snoring rates determined using SnoreClock were then compared with those obtained using the manual method. The mean values of SnoreClock’s sensitivity, specificity, negative predictive value, and positive predictive value were 78% ± 25%, 97% ± 4%, 97% ± 4%, and 65% ± 35%, respectively (Table 4). Sensitivity versus 1−specificity was plotted with a smoothing line (Fig. 3). Different points according to different sensitivities and specificities had distinct positive and negative predictive values. We carefully calculated the area under the receiver operating characteristic curve (AUC) for every recording (201 AUCs for 201 recordings). The mean value of the AUC was 0.946 ± 0.528. Thus, Snore Clock had a high predictive value for snoring.

**Table 2:** Snoring rates estimated by SnoreClock app and manual method

Variables	Results
Records enrolled for analysis	201
Records time, hours	7.0±1.1
Snoring rates by SnoreClock app, %	11.3±10.8
Snoring rates by manual method, %	11.6±12.9
Difference of snoring rates by app–manual method, %	−0.3±5.5
Absolute difference of snoring rates between app and manual method, %	3.6±4.2
No snoring, but app determined snoring, rate, %	2.6±3.2
Snoring yes, but app determined no snoring, rate, %	2.8±4.4
Snoring rates, both by app and manual method, %	8.8±10.3
No snoring, both by app and manual method, %	85.8±13.3

The results were shown as mean ± SD

**Table 3:** The associations between the variables listed and the snoring rates by SnoreClock app

Variables	Correlation	*β*	*p* value
Record time, hour	−0.021	−0.22	0.763
Snoring rate by manual method, %	0.907	0.76	<0.001
Difference of snoring rates by app–manual method, %	−0.172	−0.34	0.015
Absolute difference of snoring rates between app and manual method, %	0.302	0.78	<0.001
Snoring rates, both by app and manual method, %	0.956	1.00	<0.001
No snoring, both by app and manual method, %	−0.955	−0.78	<0.001
No snoring, but app determined snoring, rate, %	0.284	0.96	<0.001
Snoring yes, but app determined no snoring, rate, %	0.431	1.06	<0.001

**Table 4:** The mean parameters of snoring detection by SnoreClock app

Variables	Values
Sensitivity, %	78.2±24.5
Specificity, %	97.0±3.6
False negative, %	21.8±24.5
False positive, %	3.0±3.6
Negative predictive value, %	97.0±3.6
Positive predictive value, %	65.3±35.2
AUC*, %	94.6 ± 52.8

*AUC, area under the curve (receiver operating characteristic curve)

These recordings were time-series data. Each night’s sleep data were represented on 941 ± 199 px (range 540–1125 px) on the smartphone screens. Therefore, 1 px represented 28.0 ± 7.8 s. The app displayed the time bands of snoring on the screen. Manual snoring bands could also be drawn. We compared these snoring bands for every record. The chance of making a type II error (i.e., false-negative rate) is known as beta (β). We examined the false-negative rate for every recording. The power, represented by 1 − β, was acquired for every recording, and the mean power in our study was 97% ± 4%. The mean differences in snoring rates between the measurements by SnoreClock and estimation from the manual method for different phone brands were within ±5%. The *p* value of ANOVA for these mean differences between the iOS group and non-iOS group was 0.802.

## Discussion

In the current study, we used the variable snoring rate (i.e., snoring duration or sleep duration) to determine Snore Clock’s accuracy. A strong correlation was observed between the results obtained through a manual measurement of snoring and Snore Clock, which were reviewed by an ENT specialist. The correlation between SnoreClock and the manual method for detecting snoring was 0.907 (*p* < 0.001).

The results indicated that SnoreClock is a highly accurate app for detecting snoring. The findings also revealed that the snoring rate was significantly underestimated if SnoreClock indicated a higher snoring rate.

The positive predictive value for snoring was 65% ± 35% in the current study; elsewhere, the results have been 70% [22] and 93–96% [29]. In Shin’s study [22], researchers detected snoring using a smartphone app at users’ homes. Non-snore noises are more common at home, which affected both the present and Shin’s study; such noises were less of a concern in Camacho’s study [29], which was conducted in a hospital. Non-snore noises in patients’ sleep areas, such as bedding noise, coughing, and environmental noise [32], might be the causes of false-positive snoring recorded in this study. In addition, in their study, Camacho used the app Quit Snoring and compared the results with in-laboratory PSG data. However, they used data from only two patients undergoing in-laboratory PSG and one case study and set 53 dB as the snoring threshold in Quit Snoring. In the current study, we used snoring rate during sleep time as a variable for analysis because the snoring event is actually a period.

Another novel finding of the current study is that snoring rate might be underestimated if the snoring rate percentage is higher in SnoreClock. A similar result was also obtained in the study of Kreivi, where researchers recorded snoring with an MP3 recorder [33]. One reason for this might be that the sensitivity was set to a higher level to increase snoring detection [27]. Another reason might be the different frequencies of snoring to which the app was set.

## Limitations

This study has limitations. First, snoring apps might be unable to distinguish between the snores of different people in a room. Second, the accuracy of Snore Clock was validated by an ENT specialist, not through PSG testing, despite PSG being the gold standard method to diagnose snoring. Third, the app might be unable to determine snoring when the sounds have low volumes or their frequencies are similar to those of the background. Fourth, the recording time represented sleep duration. The definite timing of falling sleep and waking up could not be detected by Snore Clock. Fifth, we experienced difficulty in investigating the hardware with regard to the bandwidth, amplification, and filter settings. These settings may have influenced the snoring detection output. Finally, all the patients in the present study were middle aged (30–52 years), implying that the results cannot be extrapolated to other age groups.

## Conclusion

The mean accuracy rate for snoring detection by SnoreClock was 95%. The correlation between SnoreClock and the manual method for detecting snoring was strong. SnoreClock can be used on various smartphone brands and has a high predictive value for snoring. Our results validate at-home snoring detection through SnoreClock.

## Abbreviations

App: application;
AUC: area under the curve (receiver operating characteristic curve);
BMI: body mass index;
dB: decibel;
ENT: ear, nose, and throat;
HSATs: home sleep apnea tests;
Hz: hertz;
OSA: obstructive sleep apnea;
PSG: polysomnography
